# Sonocrystallization approach in crystal habit modification for improving the pharmaceutical properties of Rivaroxaban

**DOI:** 10.1016/j.ultsonch.2026.107921

**Published:** 2026-06-09

**Authors:** Maan Singh, Madhukiran R. Dhondale, Ashish K. Agrawal, Dinesh Kumar

**Affiliations:** Pharmaceutical Solid-State Research Laboratory (PSSRL), Department of Pharmaceutical Engineering and Technology, Indian Institute of Technology (BHU), Varanasi 221005, India

**Keywords:** Crystal engineering,sonocrystallization, Crystal habit modification, powder flowability, crystal size distribution (CSD), Compressibility-Tabletability-Compactability (CTC), Surface chemistry, hydrophilicity, Surface free energy

## Abstract

Poor pharmaceutical material properties of active pharmaceutical ingredients (APIs) can result in suboptimal manufacturing and therapeutic outcomes. Rivaroxaban (RIV) was explored as a model API due to its poor pharmaceutical properties and challenging industrial processability. In this research, crystal habit modification (CHM) of RIV was explored via solvent evaporation, cooling crystallization, and sonocrystallization methods. RIV (irregular) habit was modified to cuboidal, plate, and fiber via solvent evaporation; however, these were not further explored due to their large crystal sizes (>400 µm), which could pose serious dissolution and dose uniformity issues. Cooling crystallization method yielded plate (RIV_ACT) and blade-shaped (RIV_ACN) crystals with sizes < 200 µm. Further, CHM by sonocrystallization yielded tabular (RIV_SN6) crystals < 10 µm in size, which also exhibit a lower aspect ratio, a lower span value, and a narrower crystal size distribution than RIV_ACT and RIV_ACN. No polymorphic changes in modified habits were observed, as confirmed by PXRD, DSC, and TGA. Powder flowability improved significantly in the RIV_SN6 than in RIV_ACT and RIV_ACN (p < 0.0001) due to its narrow crystal size distribution. Interestingly, the RIV_SN6 showed improved tensile strength (p < 0.05) at a compression force of 300 MPa and exhibited improved wettability due to the exposure of polar functional groups on the modified habits. Furthermore, significant improvements in polar energy (p < 0.05) and ∼1.88 fold of IDR (p < 0.0001) were observed in RIV_SN6 compared to the RIV_ACT and RIV_ACN due to the prominence of polar groups and hydrophilic components on the particular facet.

## Graphical abstract

**Figure:**
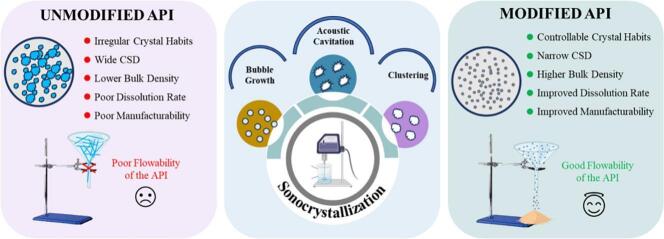

## 1 Introduction

Deep Vein Thrombosis (DVT) is a clinical complication in which a blood clot forms in the leg blood vessels. These clots reach the lungs and lodge in the pulmonary arteries, resulting in a condition known as pulmonary embolism (PE). Sometimes, clots block the supply of blood to the vital organs and result in sudden death. About 10 million DVT cases are reported every year worldwide, with 30 % mortality [1], [2], [3]. The active pharmaceutical ingredients (APIs), such as rivaroxaban (RIV), are used to treat DVT but have limitations in pharmaceutical properties, including poor aqueous solubility, a poor dissolution rate, poor flowability, poor compressibility, and limited bioavailability [4], [5], [6], [7].

To date, several approaches have been reported to address these limitations, including micronization, amorphous solid dispersion, salt formation, and modification of crystal habit by adding additives such as surfactants and polymers, as well as the addition of solubilizers and glidants [8], [9], [10], [11]. The approaches mentioned above have their own limitations, such as in the case of micronization, where an electrostatic charge can be developed, which causes poor API flowability [12]. In the instance of amorphous solid dispersion (ASDs), most polymers have the moisture absorption ability, which may result in phase separation and conversion from amorphous to crystalline [13]. The salt approach is limited only to ionizable drugs and also shows moisture instability [14]. Crystal habit modification by additives involves the extra addition of excipients [15]. Similarly, glidants and solubilizers are additional components added to the API blend to improve the powder flowability and aqueous solubility of the problematic API, respectively. These approaches cannot be applied universally to each API to improve the pharmaceutical properties. Primarily, granulation is performed to enhance the flowability and compressibility of the API blend prior to tablet compression. This granulation process requires the addition of excipients, such as diluents, binders, and glidants, as well as specific instruments, including a v-blender (used for mixing the blend before granulation) and a fluidized bed dryer [16], [17], [18]. This makes granulation expensive and time-consuming.

**Fig. 1:**
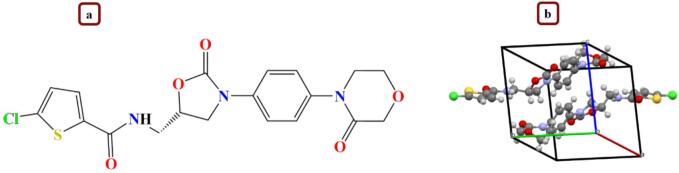
Structure of Rivaroxaban in a) 2D and b) 3D.

Most APIs exist as non-isotropic crystals, such as needles, generally exhibiting poor micromeritics and physicochemical properties. The isotropic crystals, such as cubical and spherical, are the preferred crystal habits as they show better micromeritics and physicochemical properties [19], [20], [21], [22]. CHM alters its exterior shape without altering its inner structure, making it an ideal technique for improving pharmaceutical properties [23]. The CHM experimentally attained by varying the solvent media, solvent-antisolvent ratio, optimizing the degree of saturation, addition of polymer, etc. Crystal engineering (CE) is an advanced technique that can effectively enhance the pharmaceutical properties of APIs by modifying crystal habit through sonocrystallization. Sonocrystallization (SONO) is an ultrasound-based technology used to acquire a desired crystal habit, crystal size, and crystal size distribution (CSD). The mechanisms for sonocrystallization are stated as (a) the collapse of bubbles produced by acoustic cavitation, (b) the size and volume of the bubble affect the nucleation rate, and (c) higher power intensity produces more bubbles and enhances nucleation [24]. The incorporation of uniform ultrasound energy enhances nucleation rates, reduces metastable zone width, induction time, and supersaturation levels, and produces smaller crystals with a uniform size distribution [25]. Due to their modified crystal habit, smaller crystal size, and narrow particle size distribution, these crystals exhibit improved flowability, higher packing density of powders, enhanced compressibility, and a faster dissolution rate [26]. Modified crystals with an isotropic habit and narrow CSD are ideal for direct compression of tablets and can simplify manufacturing by avoiding the tedious granulation process [27]. CHM by sonocrystallization depends on various factors, including solvents, sonication power, agitation speed, degree of supersaturation, and temperature [28], [29], [30], [31], [32], [33]. RIV **(**Fig. 1**a & b)** is chosen as a model API because of its poor flow properties, high PSD, poor aqueous solubility, and poor dissolution rate, which makes it an ideal API for performing CHM by sonocrystallization [34].

Thus, the overall aim of this research work is to explore the applicability of sonocrystallization for CHM and to examine the influence of the modified habit of the API on its pharmaceutical properties. In this study, CHM was first performed by using the conventional approach (solvent evaporation and cooling crystallization) and afterwords using sonocrystallization. The morphological evaluation was performed using scanning electron microscopy (SEM), and any polymorphic changes were confirmed by solid-state characterization techniques, comprising powder X-ray diffraction (PXRD), differential scanning calorimetry (DSC), X-ray photoelectron spectroscopy (XPS), thermogravimetric analysis (TGA), and Fourier-transform infrared (FTIR) spectroscopy. The impact of modified crystal habit on powder flow properties or micromeritic properties was studied by Carr’s index and angle of repose (AoR). Compressibility, tabletability, and compactability studies were also carried out to determine the manufacturability of the new crystal habits. Surface characterization was performed to assess wettability tendency, surface energies, surface elemental analysis, and surface area. Further, the impact of modified habits on the intrinsic dissolution rate (IDR) has also been reported. Finally, an accelerated stability study under stress conditions was performed to check its impact on the pharmaceutical properties of the API.

## 2 Materials and methods

### 2.1 Materials

The API, Rivaroxaban (RIV), with 99.6 % purity, and CAS No. 366789–02-8, was provided as a gift sample by Mylan Laboratories Limited, Hyderabad, India. Analytical grade organic solvents with purity ≥ 99 % (acetone (CAS No. 67–64-1), acetonitrile (CAS No. 75–05-8), ethanol (CAS No. 64–17-5), & chloroform CAS No. 67–66-3) and other relevant chemicals were procured by SD Finechemicals Limited, India. Ultrasonic waves were generated using the sonicator procured from Labman Scientific Instruments Pvt. Ltd., India. The stability chamber (Model: SRL/SC-10A, Company: SR Lab Instruments) was used for stability studies.

### 2.2 Experimental methods

#### 2.2.1 Polymorphic confirmation of RIV

The RIV exists in 6 polymorphs, of which polymorph I (crystalline) is in the marketed formulations. This polymorph I (crystalline) has a 4-fold higher solubility and is more stable than other forms [35], [36], [37]. Hence, a preliminary characterization of the received API sample was performed to confirm the polymorphic form using PXRD, DSC, and TGA.

#### 2.2.2 Solvent screening and saturation solubility studies

Primarily, the solvents for the solubility study were chosen on the basis of increasing order of their polarity. The saturation solubility was measured by adding an additional amount of RIV to a known amount of selected solvents in a sealed Eppendorf tube. The tube was allowed to equilibrate for 72 h in a digital shaker at room temperature. After this period, the samples were removed from the shaker, filtered, and further diluted before being analysed for their soluble content. The graph was plotted between absorbance and concentration using the standard calibration curve obtained with a UV–visible spectrophotometer (Model Cary 60, Company Agilent) [38]. The solubility study was performed in triplicate.

#### 2.2.3 CHM by the conventional methods (Solvent evaporation and cooling crystallisation)

##### 2.2.3.1 Solvent evaporation method

In Method-1, on the basis of solubility studies carried as per section 2.2.2, four solvents, such as acetone, acetonitrile, ethanol, and chloroform, were selected for the CHM. In brief, the RIV was dissolved in selected solvents (total volume: 20 ml), filtered, and then undergoes solvent evaporation at room temperature. After evaporation, the solution reached its supersaturation level, and the nucleation and further crystal growth of RIV were observed. The obtained crystals were dried and stored in closed vials for further testing [39]. The flow diagram for the above-mentioned method is shown in Fig. S**1** **in the supplementary file**.

##### 2.2.3.2 Cooling crystallization method

A cooling crystallization method was used to perform the CHM of the RIV_API using the double-jacketed glass reactor. All the experiments were performed in triplicate (detailed data provided in Figs. S**4, S5, & S6** **in the supplementary file**). In method-2, a saturated solution (total volume: 400 ml) of the RIV was prepared by adding the RIV to the selected solvent and then heating it at 60 °C in a double-jacketed glass reactor. Then, the solution was then filtered to exclude any unwanted impurities and cooled (0.5 °C/min) to −10 °C under continuous stirring at 100 rpm. During the cooling period, the crystallization of the RIV was observed. Finally, the acquired crystals were filtered, dried, and packaged in closed vials for further studies [40]. The flow diagram for cooling crystallization is shown in Fig. S**2** **in the supplementary file**.

#### 2.2.4 CHM by the novel sonocrystallization method

To further modify the crystal habit of RIV_API, sonocrystallization was performed in acetonitrile. In method-3 **(**Fig. S**3** **in the supplementary file)**, the RIV was dissolved in the selected solvents (total volume: 50 ml) under heating. The solution was then filtered and then allowed to cool at room temperature to get the supersaturated solution. Ultrasonication was then applied using a probe sonicator with a fixed frequency of 20 kHz by dipping the sonication probe into the supersaturated solution. The relevant process parameters, including ultrasonic power rate (ranging from 10 to 60), interval of ultrasonication (2 to 6 min), and pulse on/off duration (2:1), were optimized. The acquired crystals were dried and packaged in closed vials until further characterization [28]. All the experiments were carried out in triplicate (n = 3).

### 2.3 Characterization techniques

#### 2.3.1 Solid-state characterization

##### 2.3.1.1 Scanning electron microscopy (SEM)

The obtained crystals were characterized for their morphology using SEM (JCM-6000 Plus JEOL Asia Pvt. Ltd), operating in the range 10–20  kV. The crystal samples were positioned on double-sided sticky carbon tape and further sputter-coated with palladium: gold (40:60). Finally, the SEM micrographs were taken at several magnification levels to ensure crystal size and morphology.

##### 2.3.1.2 Fourier transform infrared spectroscopy (FTIR)

The crystal sample and KBr (1:100 w/w) were mixed in a mortar and pestle, then compressed into a pellet using a hydraulic press under a compression force of 3 tons (Kimaya Engineers, India). The compressed pellet was examined by FTIR instrument between the range of 4000 cm ^− 1^ to 400 cm ^− 1^ (Nicolet iS5, THERMO Scientific Instruments) to evaluate the changes in the spectral pattern of the typical peaks of the RIV and its modified crystals.

##### 2.3.1.3 Thermogravimetric analysis (TGA)

TGA was done to study the degradation pattern of RIV by measuring the variations in weight loss vs temperature. The crystal sample was load up into an alumina crucible (40 µl), with a nitrogen gas flow rate of ∼ 100 mL/min with a heating rate of ∼ 20 °C/min, over a range of 50 °C to 600 °C [28].

##### 2.3.1.4 Differential scanning calorimetry (DSC)

Thermal behaviour, enthalpy, melting point, crystallinity, and any polymorphic changes in the samples were determined by DSC, calibrated with an indium standard at 156 °C. The samples (∼5 mg) spreaded uniformly in an aluminum pan (volume 40  µl) of a DSC (Model: DSC60+, Shimadzu Pvt. Ltd.) were evaluated in the range from 20 °C to 400 °C at a ∼ 20 °C/min heating rate under a nitrogen gas flow stream (∼100  mL/min). The important and characteristic endothermic peaks of the RIV sample were analyzed and then compared with its modified crystal samples [28].

##### 2.3.1.5 Powder X-ray diffraction (PXRD)

The crystal samples thoroughly characterized to analyze any polymorphic changes in RIV and its modified crystal sample using the PXRD instrument (Miniflex 600, RIGAKU Corporation). The crystal sample was scanned (5°/min) at a step size of 0.05° over a 2θ range of 5° to 40°.

#### 2.3.2 Crystal shape, size, span value, aspect ratio, and crystal size distribution (CSD) [41], [42]

Crystal shape analyses were conducted using SEM. Crystal size was measured in the ImageJ software by uploading SEM images. The span and aspect ratio values were calculated to determine crystal size distribution (CSD) and shape uniformity, respectively (on a sample size of 100 crystals). The span value was further calculated by using **Eq.** (1)**.**

(1) $$\mathrm{Span}\;\mathrm{value}=\frac{{\text{D}}_{\text{90}}\text{-}{\text{D}}_{\text{10}}}{{\text{D}}_{\text{50}}}\text{×100}$$

The aspect ratio was calculated by using **Eq.** (2)**.**

(2) $$\mathrm{Aspect}\;\mathrm{ratio}=\frac{\text{length of the crystal}}{\text{width of the crystal}}$$

#### 2.3.3 Powder flow properties

Accurately measured crystal samples were transferred to a calibrated measuring cylinder to determine the bulk volume and tapped volume. On this basis their relative bulk and tapped densities were calculated. Bulk density was determined by pouring an precisely weighed quantity of samples into the calibrated measuring cylinder. The tapped density was measured applying a digital bulk density equipment (1250 taps, Ikon Instruments, India) [43]. Samples packing ability was measured using the compressibility index (Carr’s index), as defined in **Eq.** (3).

(3) $$\text{Car}{\text{r}}^{\prime }\text{s index}\;=\;\frac{\rho_{\text{T}}\text{-}\rho_{\text{B}}}{\rho_{\text{T}}}\text{×100}$$

where, $\rho_{B}$ is bulk density in (g/mL), and $\rho_{T}$ is tapped density in (g/mL).

The powder flowability was further measured using the angle of repose, as determined by the fixed funnel mode using the manual powder flow analyzer (Model: QMPT 01, Quality Lab Solutions, India). The amount of powder was weighed accurately and allowed to pass through the designated hole of a fixed funnel. The pile height and diameter were calculated by means of a calibrated measuring scale, and the AoR (θ) was further calculated using **Eq.** (4).

(4) $$\text{A}\text{oR (θ)=}{\text{tan}}^{\text{-1}}\frac{\text{h}}{\text{r}}$$

where ‘h’ is the pile height (cm) and ‘*r*’ is the pile radius (cm) [22]. All of the experimental measurements were carried out in triplicate [28].

#### 2.3.4 Compressibility, Tabletability, and Compactability (CTC) profiling

The ability of API crystals in powder form to shrink their volume under specifically applied pressure is generally defined by a compressibility plot. This value of compressibility is represented by the solid fraction (SF) of that particluar tablet at multiple different compression pressures, as given by **Eq.** (5).

(5) $$\;Compressibility=\frac{\text{solid fraction}}{\text{compression force}}$$

The SF is further calculated by using **Eq.** (6):

(6) $$\mathrm{SF}\;=\;\frac{\text{pellet density}}{\text{true Density}}$$

(7) $$\text{Pellet density}=\frac{\text{mass (mg)}}{\text{π×}{\text{radius}}^{\text{2}}\left(\text{mm}\right)\text{×thickness(mm)}}$$

**Equation** (7) is used to calculate the pellet density. The true density was determined using a helium pycnometer (Pycno 32, Smart Instruments, Mumbai, India) in triplicate at 25 ± 2 °C / 40 ± 5 % relative humidity.

The incorporation of compression force generally changes the powder into a robust tablet of definite tensile strength and is precise determined by the **Tabletability** plot, which plots tensile strength (TS) on X-axis against compression force on Y-axis. This tensile strength (σ) value is evaluated using **Eq.** (8):

(8) $$\sigma =\frac{\text{2x}}{\text{πdt}}$$

where “*x*” is the pellet hardness (kg/cm^2^), “*d*” is the diameter (mm), and “*t*” is the thickness (mm). Tensile strength (σ) was reported in MPa.

**Compactability** is referred to as the capability of the powdered/granular material of interest to be compressed into a tablet of specified tensile strength **(Eq.** (9)). It is derived from the plot between the SF and the TS of the pellet [44], [45], [46], [47].

(9) $$\mathrm{Compactability}=\;\frac{\text{solid fraction}}{\text{tensile strength}}$$

#### 2.3.5 Wettability using contact angle measurements

Wettability is defined as the ability of the pellet surface of the API crystal to be wetted by the liquid. The wettability can be determined by measuring the contact angle relating a sessile drop and the upper surface of the crystal pellet. Firstly, the samples were compressed into pellets using a hydraulic press (Kimaya Engineers, India) at 3-ton pressure with a 1-minute dwell time. The contact angle was then measured by the sessile drop method (Dura Vision CAM-02, India). In the contact angle study, double-distilled water (DM) was employed as a polar solvent, ethylene glycol (EG) employed as a semi-polar solvent, and diiodomethane (DIM) employed as a non-polar solvent. The crystal pellets were positioned onto the contact angle goniometer stage, and a solvent drop was dispensed using a syringe. The frames of the drop and pellet were captured using a high-speed camera after the drop stabilization (∼30–45 s after dropping). All of the contact angle experiments were performed with a sample size of n = 6 [48], [49], [50], [51].

#### 2.3.6 Determination of surface free energy

Based on the equations given by the Van-Oss-Chaudhury-Good (VCG), the total surface energy (γS), the polar energy (γAB), the acidic component (γA), and the dispersive surface energy (γLW) were calculated using previously determined contact angle values of the samples [28].

The principal **Eq.** (10) as per this theory is as follows.

(10) $$\text{(1+cosθ}{)\gamma }_{\text{LV}}\text{=2[}\sqrt{\gamma_{\text{S}}^{\text{LW}}\gamma_{\text{L}}^{\text{LW}}}+\sqrt{\gamma_{\text{S}}^{\text{A}}\gamma_{\text{L}}^{\text{B}}}+\sqrt{\gamma_{\text{L}}^{\text{A}}\gamma_{\text{S}}^{\text{B}}}\text{]}$$

where, γ_LV_ represents the surface tension, $\gamma_{S}^{\mathrm{LW}}$ represents the dispersive surface energy of a solid, and $\gamma_{L}^{\mathrm{LW}}$ stands for the dispersive surface energy, $\gamma_{S}^{A}$ stands for the acidic component of a solid, $\gamma_{L}^{B}$ stands for the basic component of a liquid, $\gamma_{L}^{A}$ stands for the acidic component of a liquid, and $\gamma_{S}^{B}$ stands for the basic component of a solid [28], [51].

#### 2.3.7 X-ray photon spectroscopy (XPS)

The relevant chemical elements prominently present on the pellet upper surface were examined by the XPS (Model: K-Alpha, Thermo Fisher Scientific) with an optimum analysis depth of ∼ 10  nm. This pellet for the XPS analysis was prepared by applying a compression force of 3 tons [28], [51], [52]. The surface polarity ratio (SPR) is used to determine the percentage hydrophilicity of the pellet surface. The chemical elements present on the surface, as determined by XPS, were used to calculate the SPR. **Equation** (11) was used to calculate the SPR [28], [51].

(11) $$\mathrm{SPR}\;=\frac{\text{percentage of total polar components}}{\text{percentage total non-polar components}}$$

#### 2.3.8 Surface chemistry by computational simulation study

The crystal structure of RIV (CSD code: 1854617) was downloaded from the Cambridge Structural Database (CSD). The identification of important crystal facets and the number of relevant functional groups available prominently on the facet surface was determined through Mercury software 2022.2.0. The peaks associated with the hkl values, along with peak intensities, were determined from the PXRD pattern using Mercury software 2022.2.0 and OriginLab software version 10.2.60.16. [28], [51].

**Determination of an important facet, PXRD peaks associated with hkl value, and peak intensities:** To determine the important facets, the CSD file was loaded into Mercury. The powder pattern was selected in the “calculate” tab, and then the peaks associated with hkl values along with their intensities were analysed. The peak intensities were manually calculated using OriginLab software [53]. Based on peak intensity, the facet list was created. The slice of the important facet was determined by manually entering the hkl values for that facet in Mercury software. The slice of the important facet, containing the exposed functional groups, was checked manually by rotating/adjusting the facet slice.

#### 2.3.9 Surface area analysis

The surface area of the samples was calculated by the nitrogen gas adsorption method using Brunauer-Emmett-Teller (BET) (BELLSORP MAX II & BELCAT-II, MicrotracBEL Corp). The crystal sample (100–200 mg) was slowly degassed for 6 h under vacuum at ∼ 100 °C. After degassing, isotherms were measured employing a fully automated BET instrument [41], [54].

#### 2.3.10 Intrinsic dissolution rate (IDR) studies

The intrinsic dissolution apparatus (Analysis Instrument, India) as per USP < 1087 > was employed to calculate IDR [55]. Accurately weighed 250 mg of each sample was compressed into pellets under a compression force (0.5 ton) over a 1-minute dwell time using a hydraulic press. Before compression to form a pellet, each crystal sample was passed through a sieve (ASTM 100 mesh) to ensure all crystal sizes were within the same range (retained original morphology: Fig. S8). So that, only the impact of crystal habit on IDR can be determined. The test was performed in 900 ml of acetate buffer (pH 4.5) + 0.4 % SLS w/w at 37 °C with 75 rpm. A ∼ 5 ml sample was taken out at predecided time intervals, and the same quantity of fresh buffer was reloaded to preserve the required sink conditions. The samples were filtered by employing a 0.2 µm nylon filter and then further analyzed at 250 nm using a validated UV method (Model: Cary 60, Agilent). Only one side of the pellet surface, having a surface area of 0.502 cm^2^, was exposed for the elution from the IDR apparatus. [56], [57], [58].

#### 2.3.11 Stress stability study

The stress stability study was performed in the stability chamber (SRL/SC-10A, SR Lab Instruments, India) by maintaining storage conditions at 40 °C and 75 % RH for 3 months. Each sample was removed from the stability chamber after 1, 2, and 3 months and analyzed by DSC and PXRD, as well for powder flow and IDR [59], [60].

#### 2.3.12 Statistical analysis

The mean ± standard deviation (SD) is used to express all the data. GraphPad Prism was used to conduct the ANOVA analysis.

## 3 Results and discussion

### 3.1 Preliminary characterization and polymorphic confirmation of RIV

**Fig. 2:**
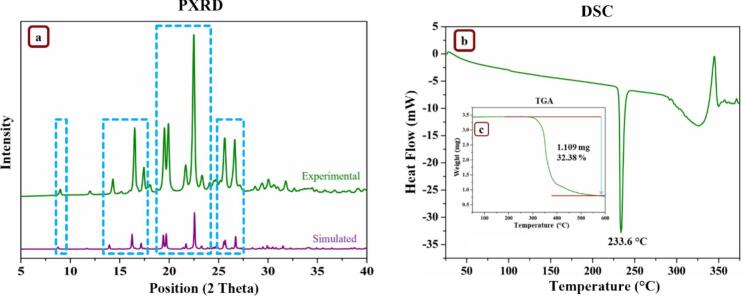
Preliminary confirmation of the RIV form I (crystalline) by **(a)** Simulated (CCDC#1854617) and experimental PXRD pattern, **(b)** DSC thermogram, and **(c)** TGA.

The preliminary characterization of the received RIV was done by PXRD, DSC, and TGA to confirm the polymorphic form of the RIV. The PXRD analysis of RIV (experimental) exhibits characteristic peaks (2θ) at 8.9°, 14.2°, 16.6°, 19.5°, 19.8°, 22.5°, 25.6°, and 27.1°. They are also present in the simulated PXRD pattern of RIV (CSD code: 1854617) **(**Fig. 2**a)**. A similar PXRD pattern confirmed that the received RIV is Form I (crystalline). This was also confirmed by DSC, which showed that the melting point of the received RIV was 233.6 °C, the same as the melting point ranging in 228–234 °C of the RIV form I **(**Fig. 2**b)** [36], [37]. Further, the TGA study was performed, which showed no weight loss of the received RIV up to its melting point. A significant change in weight was noted beyond its melting point, which indicates thermal degradation of RIV. A similar weight loss pattern has also been reported in the literature **(**Fig. 2**c)** [61]. As per the PXRD pattern, DSC, and TGA behavior, henceforth, the term ‘RIV’ refers to the RIV_API.

### 3.2 Solvent selection and saturation solubility studies

Primarily, the solvents for the solubility study were chosen on the basis of increasing order of their polarity. The saturation solubility study was performed as per the solvents listed in Table 1. Based on this saturation solubility values, further recrystallization experiments, and CHM optimizations were performed.

**Table 1:** Saturation solubility values of RIV_API at 25 °C and at 60 °C (mean ± SD, n = 3).

Solvent	Saturation solubility values (mg/mL) at 25 °C	Saturation solubility values (mg/mL) at 60 °C
Acetone	1 ± 0.24	1.5 ± 0.31
Acetonitrile	1 ± 0.22	3 ± 0.54
Ethanol	0.8 ± 0.13	1.25 ± 0.32
Chloroform	1 ± 0.18	1.5 ± 0.28
Water	Insoluble	

### 3.3 CHM by solvent evaporation method

**Fig. 3:**
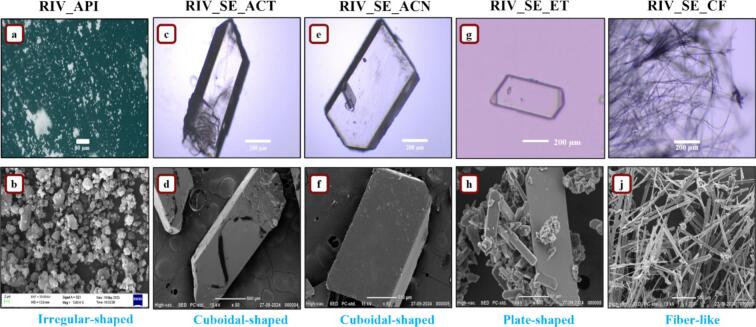
SEM images of RIV_API (a & b) recrystallized in acetone represented as RIV_SE_ACT (c & d), in acetonitrile represented as RIV_SE_ACN (e & f), in ethanol as RIV_SE_ET (g & h), and in chloroform as RIV_SE_CF (i & j).

The crystal habit of the API used for recrystallization can be clearly observed as irregular-shaped in Fig. 3**a & 3b**. The CHM of the RIV_API was performed in the selected solvents as mentioned in Table 1. All the solvent evaporation based experiments were performed in triplicate (n = 3). The CHM in acetone **(3c & 3d)** and acetonitrile **(3e & 3f)** yields cuboidal-shaped crystals, whereas in ethanol **(g & h)** and chloroform **(i & j)**, it results in plate-shaped and fiber-like crystals, respectively. It can be evidently visible that the crystal habit of the RIV_API in all the solvents was successfully changed from irregular shaped crystals to cuboidal, plates, and fiber-like crystals.

CHM was observed in all the solvents; however, the average crystal size was too large **(details given in** Table 2**).** Due to the formation of larger crystals in acetone, acetonitrile, and ethanol. Method 1 was excluded and not considered for further studies. Too large crystals (>400 µm) have the possibility of a poor dissolution pattern due to a smaller surface area. In addition, these large crystals also exhibit poor dosage uniformity because the API dose is low, and a few large crystals can cause significant variations in the amount of API per tablet, leading to inconsistent dosing and reduced patient safety [62]. On the other hand, chloroform was not considered for further CHM experiments due to the formation of fragile, fiber-like crystals having a high aspect ratio value, which are prone to breaking easily [63], [64]. Only acetone, acetonitrile, and ethanol were considered for further CHM experiments, as clear CHM was observed, and process optimization can bring CSD in the desirable range.

**Table 2:** Summary of CHM experiments using the solvent evaporation method.

Solvents	Crystal Habit	Average Crystal Size (µm)	Inference
Acetone	Cuboidal-shaped crystals	825.4	Habit modified with a large crystal size
Acetonitrile	Cuboidal-shaped crystals	727.5	Habit modified with a large crystal size
Ethanol	Plate-shaped crystals	413.6	Habit modified with a large crystal size
Chloroform	Fiber-like crystals	910.3	Habit modified with a large crystal size

### 3.4 CHM by cooling crystallization method

**Fig. 4:**
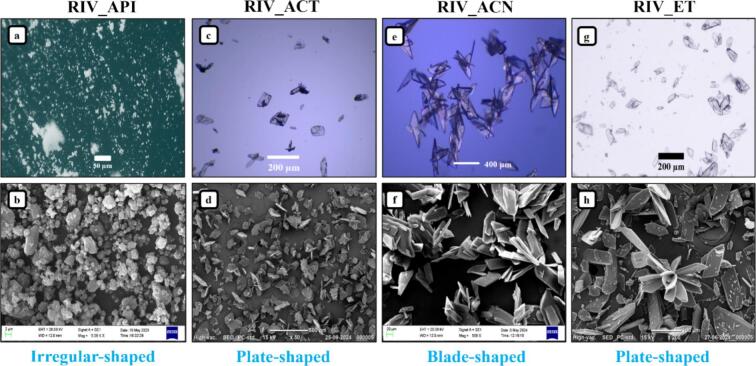
Optical microscopic and SEM images of RIV_API (a & b) recrystallized by the cooling crystallization method in acetone, represented as RIV_ACT (c & d), in acetonitrile, represented as RIV_ACN (e & f), and in ethanol, represented as RIV_ET (g & h).

The CHM of the RIV_API **(**Fig. 4**a & 4b)** yielded plate-shaped crystals from irregular-shaped crystals in acetone (RIV_ACT) and exhibited a similar habit with uniform crystal size **(**Fig. 4**c & 4d)**. The crystal habit obtained in acetone by using method-2 was considered hereafter as the main habit on the RIV.

**Fig. 5:**
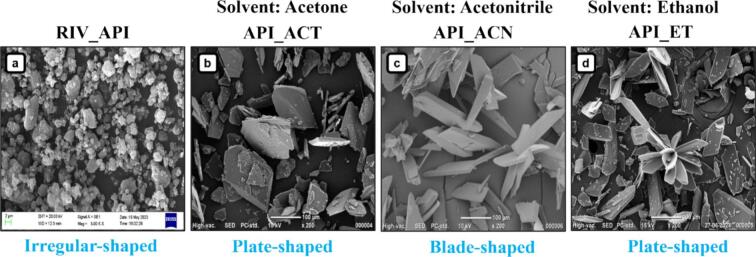
SEM images of RIV crystals represented as **(a)** RIV_API, **(b)** API_ACT, **(c)** API_ACN, and **(d)** API_ET.

Similarly, acetonitrile (RIV_ACN) yielded modified crystal habits from irregular-shaped crystals to blade-shaped crystals with uniform crystal size **(**Fig. 4**e & 4f)**. Further, ethanol (RIV_ACT) yielded plate-shaped crystals with uniform sizes **(**Fig. 4**g & 4 h)**. Thus, the cooling crystallization modified irregularly shaped crystals to a plate and blade-shaped crystal, with a uniform crystal size distribution (Method-2). The variable polar nature of the solvents used in the recrystallization of API can be a possible reason for the CHM. In general, the polar or non-polar groups present in the solvents probably interact with the prominent surface functional groups of the APIs based on its relative polarity [28]. Further, based on their solvent polarity, the API crystals are allowed to grow along one or more axes. If the interaction of the solvent with the API along one axis is greater, then it leads to faster growth, and the resulting habit could be needle-shaped. However, if crystal growth happens in two or more than two axis, the crystal habit generally shifts from needle to other different shapes (blade, tabular, rod, plates, etc.). Hence, the formation of plate and blade-shaped crystals was due to the interaction of the polar and non-polar groups of solvent with the functional groups of the API along more than one axis [59], [65], [66]. This phenomenon was also observed in CHM experiments, where changing the solvent type altered the habit due to the presence of polar or non-polar components in the selected solvent. The combined data of all the crystal habits obtained by Method-2 are shown in Fig. 5.

The crystal habit obtained in RIV_ACT was considered the main habit of the RIV, and in comparison to that, only RIV_ACN showed a modified crystal habit (blade-shaped crystals). As no habit modification was observed in RIV_ET via cooling crystallization. It was excluded from further studies. Hence, three systems (RIV_API, RIV_ACT, and RIV_ACN) have been selected for further studies. In method-2, CHM was clearly observed in acetonitrile, so sonocrystallization was performed only in acetonitrile [67].

### 3.5 CHM by sonocrystallization

**Fig. 6:**
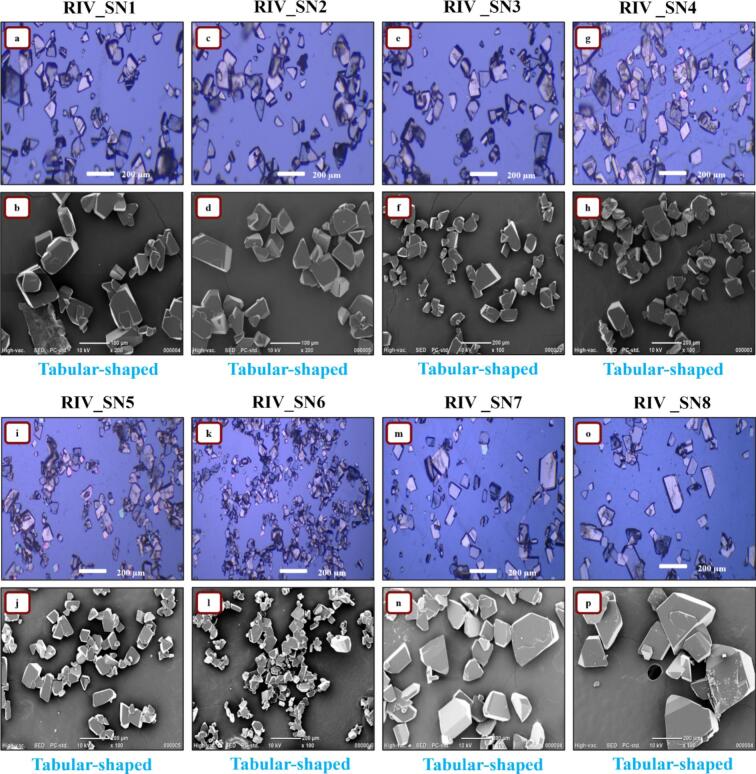
Optical microscopic and SEM images in acetonitrile using ultrasonication by varying the total duration (2 min, 4 min, 6 min) and power rate (10 W for each) for the samples RIV_SN1 (a & b at 200x), RIV_SN2 (c & d at 200x), RIV_SN3 (e & f), respectively and by varying total duration (6 min for each) and power rate (20 W, 30 W, 40 W, 50 W, 60 W) for the samples RIV_SN4 (g & h), RIV_SN5 (i & j), RIV_SN6 (k & l)**,** RIV_SN7 (m & n), and RIV_SN8 (o & p).

Firstly, CHM was performed by varying the total duration (in minutes) of sonication at a 10 % sonication power rate. It was found that all three sonocrystallized samples (RIV_SN1, RIV_SN2, and RIV_SN3) yielded tabular-shaped crystals **(**Fig. 6**)**. Interestingly, it was observed that the crystal size, as represented by D_90_, of the sample decreased with increasing sonication time **(**Table 3**)**.

**Table 3:** Sonication run parameters (Run time: 2 sec & Elapsed time: 1 sec), crystal size distribution (CSD), habit, span value, and aspect ratio of sonocrystallized crystals. All values reported (Mean ± SD, n = 100).

Sample Name	Total Duration (min)	Power Rate (%/W)	Crystal Habit	D_10_ (µm)	D_50_ (µm)	D_90_ (µm)	Span Value	Aspect Ratio	**Sample Recovery (%)**
RIV_SN1	2	10/65	Tabular	90.6 ± 1.31	112.7 ± 1.23	203.3 ± 1.24	1.00 ± 0.13	1.46 ± 0.27	∼95
RIV_SN2	4	10/65	Tabular	84.3 ± 1.53	99.1 ± 1.54	187.4 ± 1.47	1.04 ± 0.11	1.48 ± 0.23	∼95
RIV_SN3	6	10/65	Tabular	75 ± 1.81	88 ± 1.98	168.7 ± 1.45	1.06 ± 0.17	1.51 ± 0.36	∼95
RIV_SN4	6	20/130	Tabular	55.1 ± 1.14	64.3 ± 1.65	117.8 ± 1.56	0.98 ± 0.11	1.32 ± 0.33	∼95
RIV_SN5	6	30/195	Tabular	26 ± 1.32	43.3 ± 1.99	72.2 ± 1.67	1.07 ± 0.21	1.45 ± 0.31	∼95
**RIV_SN6**	6	40/260	Tabular	2.4 ± 0.98	5.5 ± 1.11	**7.1 ± 1.24**	**0.85 ± 0.09**	1.38 ± 0.58	∼95
RIV_SN7	6	50/325	Tabular	121 ± 1.63	192 ± 2.11	386 ± 1.90	1.38 ± 0.16	1.32 ± 0.32	∼95
RIV_SN8	6	60/390	Tabular	133 ± 1.78	148 ± 2.43	394 ± 2.15	1.76 ± 0.19	1.41 ± 0.24	∼95

RIV_SN1 has a higher D_90_ (µm) value (203.3 ± 1.24) compared to RIV_SN2 (187.4 ± 1.47) and RIV_SN3 (168.7 µm ± 1.45 µm). The order of crystal size with respect to the D_90_ value was: RIV_SN1 > RIV_SN2 > RIV_SN3. Hence, it is confirmed that increasing the sonication time results in a decrease in crystal size [68]. Therefore, further sonication was performed by varying the sonication power rate from sample RIV_SN4 to RIV_SN8, while keeping the total duration of sonication at 6 min, to check its impact on crystal habit and size **(**Table 3 **&** Fig. S**7** **(provided in the supplementary file))**. It was observed that modified-shaped crystals (tabular) were obtained in all samples from RIV_SN4 to RIV_SN8 **(**Fig. 6**)**. However, the D_90_ values decreased as the power rate increased up to 40 %. As the power rate increased, more small nuclei formed, which could lead to the formation of smaller crystals [68].

On the other hand, for the power rates of 50 % & 60 %, the D_90_ value increased for the modified-tabular shaped crystals (RIV_SN7 and RIV_SN8) **(**Table 3 **&** Fig. S**7** (provided in the supplementary file)**)**. The possible reason for the increase in the crystal size (D_90_) could be due to the formation of a large number of very small nuclei and their fragments. These small-sized nuclei might have higher surface energy and an attractive force (such as the van der Wall force) compared to nuclei obtained at a power rate of up to 40 %, which could bring them closer and turned out in the creation of large crystals again (Ostwald growth). However, up to a power rate of 40 %, this energy and attractive force could be insufficient to bring them closer to form large crystals [69], [70]. Another possible reason for the increase in crystal size above 40 % power rate could be the generation of local heat induced by the high-intensity ultrasonication. The high-intensity ultrasonication produces small bubbles, and when these collapse due to acoustic cavitation, they generate extreme local heat that redissolves the small crystals (Ostwald ripening), decreases the nucleation rate, and promotes recrystallization on existing crystals [24], [71]. In addition, the increasing ultrasonic power rate destroyed or broke the generated nuclei through collisions, increasing surface activity and making them more prone to agglomeration, resulting in larger crystals [72], [73]. RIV_SN6 was chosen for further experiments on the basis of the smallest D_90_ (µm) value (**7.1 ± 1.24**) and span value (**0.85 ± 0.09**).

**Fig. 7:**
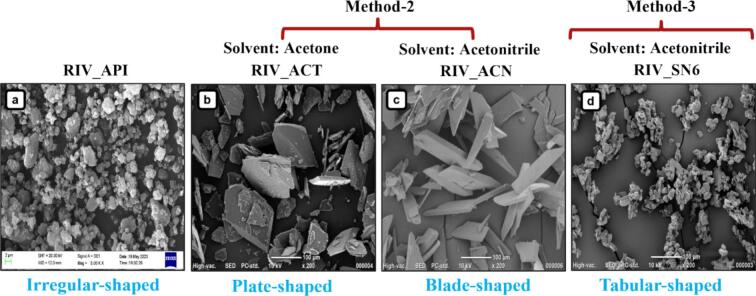
SEM images of modified RIV crystal habits **(a)** RIV_API, **(b)** RIV_ACT, **(c)** RIV_ACN, and **(d)** RIV_SN6.

Overall, method-2 yielded a plate-shaped crystal in acetone (RIV_ACT), blade-shaped crystals in acetonitrile (RIV_ACN), and method-3 yielded tabular-shaped crystal habits in acetonitrile (RIV_SN6) **(**Fig. 7**)**. Further studies were conducted on these selected samples (RIV_API, RIV_ACT, RIV_ACN, and RIV_SN6) to explore the impact of CHM on their solid-state behaviour, powder flow properties, and CTC profiling, respectively.

### 3.6 Crystal shape, size, span value, aspect ratio, and crystal size distribution (CSD) analysis

The sonocrystallization had an intense influence on crystal shape, size, span value, aspect ratio, and crystal size distributions **(**Table 4**)**.

**Table 4:** Crystal size distribution (CSD), habit, span value, and aspect ratio of RIV_API and its modified crystals. All values reported (Mean ± SD, n = 100).

Sample Name	Crystal Habit	D_10_ (µm)	D_50_ (µm)	D_90_ (µm)	Span Value	Aspect Ratio
RIV_API	Irregular-shaped	0.8 ± 0.06	1.7 ± 0.12	3.6 ± 0.87	1.66 ± 0.25	1.20 ± 0.25
RIV_ACT	Plate-shaped	66.46 ± 1.35	103.4 ± 1.43	163.17 ± 1.23	0.94 ± 0.13	1.49 ± 0.42
RIV_ACN	Blade-shaped	42.45 ± 1.12	75.6 ± 1.65	172.05 ± 1.53	1.77 ± 0.12	2.69 ± 0.21
RIV_SN6	Tabular-shaped	2.4 ± 0.98	5.5 ± 1.11	7.1 ± 1.24	0.85 ± 0.09	1.38 ± 0.58

**Fig. 8:**
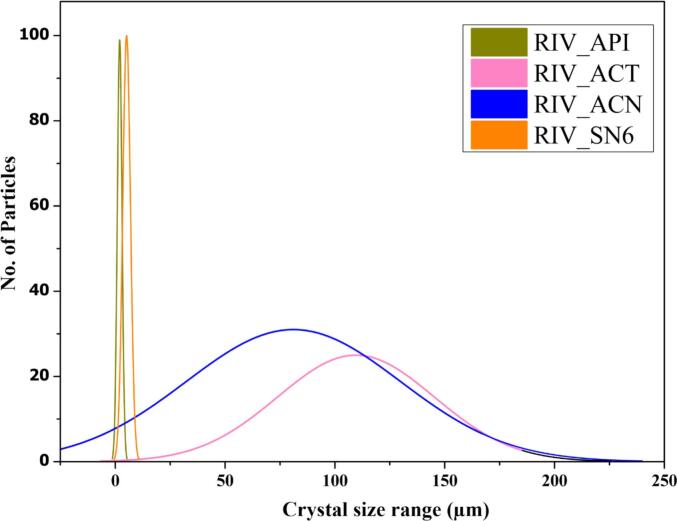
Crystal size distribution (CSD).

The D_90_ values of RIV_API, RIV_ACT, RIV_ACN, and RIV_SN6 are 3.6 ± 0.87 µm, 163.17 ± 1.23 µm, 172.05 ± 1.53 µm, and 7.1 ± 1.24 µm, respectively. The lower D_90_ of RIV_SN6 indicates maximum size reduction among other samples, such as RIV_API, RIV_ACT, and RIV_ACN. Interestingly, the span value (0.85 ± 0.09) was also lower in RIV_SN6, indicating a narrower CSD **(**Fig. 8**)** and better shape uniformity, compared to other samples (RIV_API, RIV_ACT, and RIV_ACN). The lowest aspect ratio (1.38 ± 0.58) of RIV_SN6 compared to RIV_ACT and RIV_ACN was attributed to ultrasonication, which delivers consistent energy during the crystallization process [65]. Interestingly, the aspect ratio of RIV_SN6 was higher compared to RIV_API, which could be due to its tabular-shaped crystals. It is a well-recognised notion that tabular-shaped crystals have a higher aspect ratio compared to irregularly shaped crystals, while lower compared to plate and blade-shaped crystals [74], [75].

### 3.7 Solid-state characterization

**Fig. 9:**
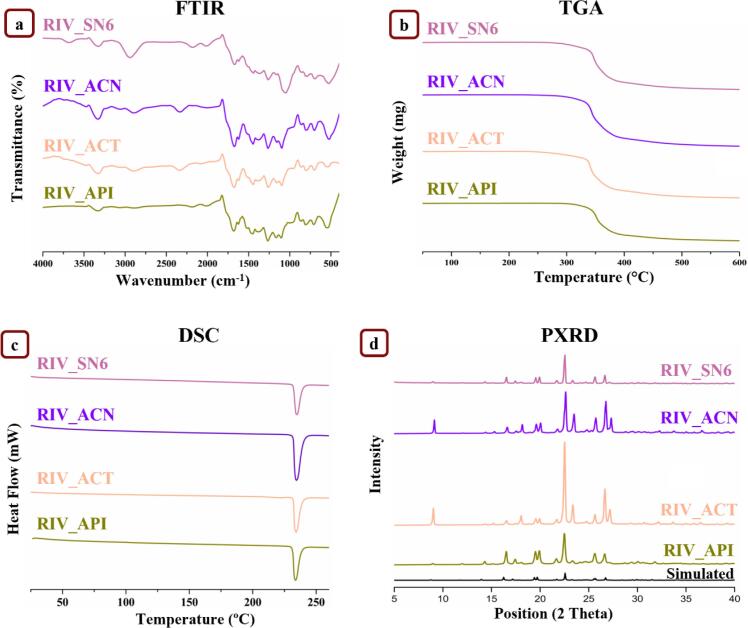
**A)** FTIR spectrums, **b)** TGA thermograms, **c)** DSC patterns, and **d)** PXRD data.

The characterization of the selected samples was performed to assess any solid-state transition during crystallization. In FTIR spectroscopic analysis **(**Fig. 9**a),** the representative peaks are linked with relevant functional groups of RIV_API at 3354 cm^−1^ for N–H amide stretches, 1734 cm^−1^ for C=O carbonyl stretches, 1516 cm^−1^ for C=C aromatic stretching, 1650 cm^−1^ for C=O stretches, 1214 cm^−1^ for C-N stretches, and 1122 cm^−1^ for S=O stretches. The peaks in FTIR spectra of the RIV_ACT, RIV_ACN, and RIV_SN6 were identical to the RIV_API **(**Fig. 9**a)**. The identically comparable spectrum revealed that there was no observable differences in their molecular structure when interacting with sonication waves and CHM in several solvents [76].

The thermal behaviour pattern of crystals was examined using TGA and DSC. RIV_API demonstrated no weight reduction up to its melting point (233.6 °C). There was significant reduction in weight was noted beyond its melting point, which indicates thermal degradation of RIV_API. The TGA pattern of modified crystals in Fig. 9**b** was on the same line as that of RIV_API, which indicates the absence of any polymorphic changes.

The DSC analyses **(**Fig. 9**c)** also revealed no significant changes in the melting points of RIV_API, RIV_ACT, RIV_ACN, and RIV_SN6, which were found to be 233.6 °C, 233.9 °C, 234.1 °C, and 233.8 °C, respectively. This reveals no polymorphic modifications in the RIV_API and its modified crystal samples.

The PXRD analysis of RIV_API **(see** Fig. 9**d)** exhibits characteristic peaks at 8.9°, 14.2°, 16.6°, 19.5°, 19.8°, 22.5°, 25.6°, and 27.1° positions (2θ), these peakswere also existent in PXRD pattern of RIV_ACT, RIV_ACN, and RIV_SN6. No changes in the diffraction peak pattern of RIV_API and other altered crystal samples imply the absence of any polymorphic change.

The peak pattern of the crystal habits remain similar if no polymorphic changes are observed. However, the intensity of the peaks can be different among different habits because of anisotropy [77]. Changes in peak intensity due to changes in the fecet area were confirmed when compared with the simulated PXRD pattern. The CSD file (CSD code: 1854617) for the RIV form I was downloaded from the CCDC database. Important facets were determined by using the Mercury software. Important facets of RIV_API are (001), (1–10), (021), (022), (120), (10–2), (032), and (11–2) **(**Fig. 9**d)**. The changes observed in the peak intensity of all the samples **(**Fig. 9**d)** were due to changes in the facet area of the modified habits [22], [77].

### 3.8 Powder flow properties

The bulk, tapped density, AoR, and Carr’s value were determined to investigate their impact on the flowability and compressibility of the API. The changes in the crystals also have a significant impact on the compressibility of the API. In general, the poor flowability of the API increases the likelihood of blocking the API blend inside the hopper during tablet compression, which can lead to inconsistencies in the dosage form [7]. A crystal habit with better flow can overcome this issue. It is reported that modified crystal habit with tabular-shaped crystals exhibits better flow compared to plate, blade, or irregularly shaped crystals [22].

**Fig. 10:**
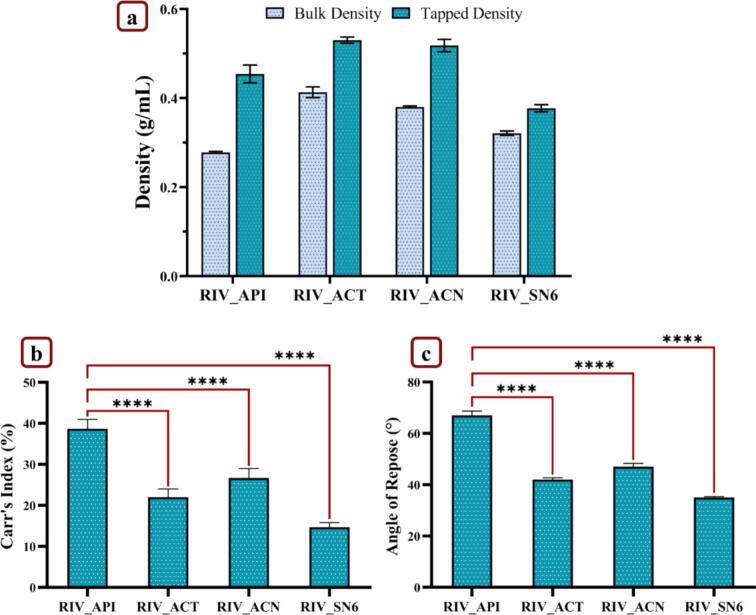
**(a)** Bulk density, tapped density, **(b)** Carr’s values, and **(c)** AoR of RIV_API and RIV_ACT, RIV_ACN, and RIV_SN6. All data values here shown as mean value ± SD (n = 3). On the basis of One and two-way ANOVA, the statistical difference is shown as *** if p < 0.001, & **** if p < 0.0001.

As reported in the literature, RIV_API exhibits poor flowability due to irregular-shaped crystals [7]. This is evident in the case of bulk density **(**Fig. 10**a)**, where RIV_API has a lower bulk density (g/ml) value (0.278 ± 0.002) than RIV_ACT of 0.413 ± 0.012 with p < 0.0001, RIV_ACN of 0.380 ± 0.002p < 0.0001, and RIV_SN6 of 0.321 ± 0.005p < 0.001. In general, large crystals have a higher density than smaller ones. The possible reason for the low bulk density of the RIV_SN6 in comparison to RIV_ACT and RIV_ACN could be due to the smaller size of the crystals with a higher specific surface area, which could lead to an increase in interparticle forces, such as van der Waals forces. These cohesive forces prevent dense settling, resulting in more fluffy, void spaces that could ultimately decrease the bulk density. The low bulk density resulted in poor flowability because the irregularly shaped crystals have a poor packing arrangement, not fitting together closely. On the other hand, RIV_ACT, RIV_ACN, and RIV_SN6 have higher bulk density compared to RIV_API, indicating an improved arrangement of crystals upon transferring into the vessel due to their crystal habit, and showed improved flowability [43]. This is also supported by Carr’s index and AoR **(**Fig. 10**b and 10c)**, which show that all the modified crystal samples, such as RIV_ACT, RIV_ACN, and RIV_SN6, exhibit significant improvements (p < 0.0001) in compressibility and flowability, respectively (Table S**1**).

### 3.9 Compressibility, Tabletability, and Compactability (CTC) of tablets

It’s crucial in the pharmaceutical industry for understanding how powders behave under compression to form tablets. This helps to determine the compression force, tensile strength, compaction pressure, porosity level, or solid fraction, needed to formulate the tablet. By understanding these parameters, tablet deformation, such as capping, chipping, lamination, picking, and sticking issues, can be easily detected at an early phase [46]. The compressibility profile provides information on how the solid fraction of the crystals increases as a compression force is applied, indicating crystal rearrangement or densification. This profile is plotted between the solid fraction and the compression force.

**Fig. 11:**
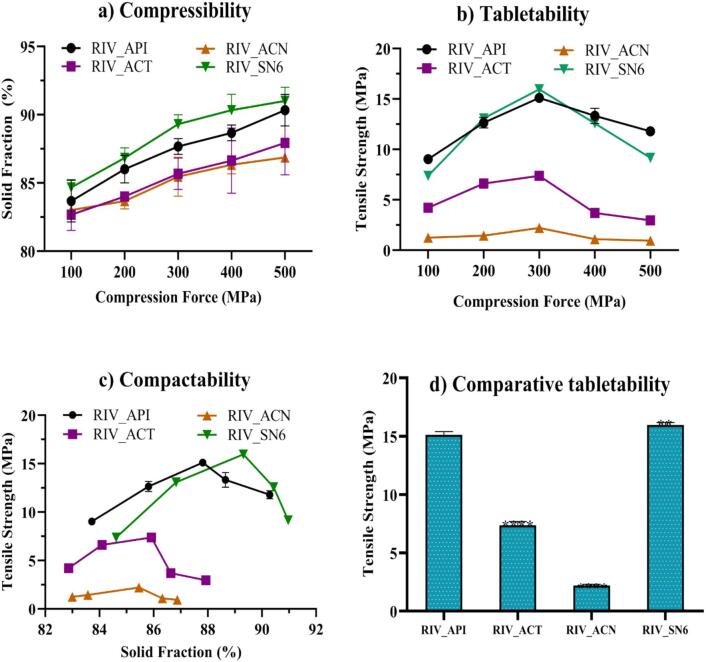
**(a)** Compressibility plot, **(b)** Tabletability plot, **(c)** Compactability plot, and **(d)** Comparative tabletability values at 300 MPa. Statiscical comparison was done with RIV_API as the control group (** if p < 0.05 and **** if p < 0.0001).

As shown in Fig. 11**a**, RIV_SN6 exhibits the maximum compressibility across the compression forces between 100 MPa and 500 MPa, compared to other modified crystal samples. Interestingly, RIV_API showed better compressibility than RIV_ACT and RIV_ACN, but was still lower than RIV_SN6. The compressibility of the crystals indicates the generation of maximum true contact between the crystals by adhesive and cohesive forces, mechanical interlocking, and van der Waals forces. The compressibility of the RIV_SN6 is higher because the CSD is much narrower in RIV_SN6.

**Fig. 12:**
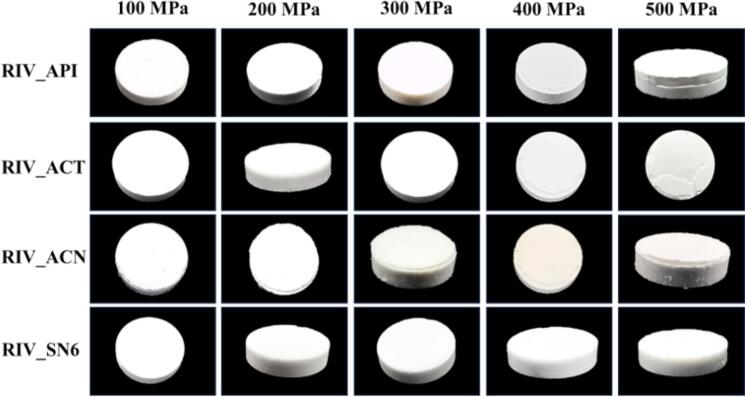
Representative pellet images of RIV_API, RIV_ACT, RIV_ACN, and RIV_SN6 at the compression force of 100 MPa to 500 MPa.

Fig. 11**b** illustrates the tabletability profile in relation to tensile strength and compression force. This profile helps to understand how well the crystals form strong tablets at a specific pressure. The RIV_SN6 showed maximum tensile strength at the 300 MPa. Further increases in the force cause decline in its tensile strength value, but do not result in any pellet deformity. RIV_API and RIV_ACT have shown a good tabletability profile up to 300 MPa. Further increases in the compression force lead to **capping and lamination.** RIV_ACN had very low tensile strength and failed due to exhibiting capping and lamination issues at all compression forces **(**Fig. 12**)**. The improved tabletability profile of the RIV_SN6 could be due to changes in crystal habit and a narrower CSD. Variation in crystal habit and CSD alters the surface area available for inter- and intraparticular interactions, potentially improving the tabletability profile.

In Fig. 11**c, a** higher bonding strength (BS) was observed in RIV_SN6 at solid fractions between 86 % and 89.5 %, which can be considered the ideal range. Below this range, pellets crumble, and above this range, capping and lamination take place [28], [78]. The narrower CSD and lower aspect ratio have a strong bonding strength ability that could lead to improved compactability of RIV_SN6. On the whole, the tabletability ranked as RIV_SN6 > RIV_API > RIV_ACT > RIV_ACN **(**Fig. 11**d)**.

This confirmed that the tabular-shaped crystals observed in RIV_SN6, with a narrow CSD, showed a good CTC profile in terms of compressibility, tabletability, and when compared to irregular (RIV_API), plate-shaped (RIV_ACT), and blade-shaped (RIV_ACN) crystal habits. The tabular crystal habit, smaller size of the crystals, and the narrow CSD, which probably leads to higher interaction and enables interparticular and intraparticular level bonding and better packing arrangement of the crystals, respectively [79], [80], [81], [82].

In Fig. 12, it is clearly observed that RIV_SN6 exhibits no tablet deformities at compression forces ranging from 100 MPa to 500 MPa. However, other samples exhibit tablet deformities, including capping and lamination. Hence, based on the CTC profile, a compression of 300 MPa is considered as a model compression force for the tablets, providing maximum tensile strength.

### 3.10 Wettability and surface free energy studies

Wettability is defined as the ability of the pellet upper layer to be wetted by the liquid. The wettability of the modified crystal samples can be changed due to the differential exposure of functional groups on their facets [83]. The amount of the polar/non-polar component can also vary due to the variation in the surface area by their crystal size reduction [83]. As given in Table 5, the contact angles with water (double-distilled water) were RIV_API (54.07 ± 0.90), RIV_ACT (65.28 ± 2.33), RIV_ACN (61.96 ± 1.79), and RIV_SN6 (49.94 ± 1.44). The order of their contact angle was RIV_ACT > RIV_ACN > RIV_API > RIV_SN6. The similar order in the contact angle with ethylene glycol was observed as RIV_ACT (42.35 ± 3.84) > RIV_ACN (34.12 ± 2.81) > RIV_API (24.39 ± 0.33) > RIV_SN6 (21.35 ± 2.31). This decrement in the contact angle with water and ethylene glycol reveals the higher wettability of the modified crystal habit pellet surface with water and ethylene glycol. A statistical significant decrease in its contact angle (****p < 0.0001) was observed in RIV_SN6 when it was in contact with water and EG.

**Table 5:** Contact angle data (mean ± SD, n = 3).

Sample Name/Contact angle values	Water (°)	EG (°)	DIM (°)
RIV_API	54.07 ± 0.90	24.39 ± 0.33	26.85 ± 0.52
RIV_ACT	65.28 ± 2.33	42.35 ± 3.84	25.73 ± 0.94
RIV_ACN	61.96 ± 1.79	34.12 ± 2.81	26.32 ± 2.84
RIV_SN6	49.94 ± 1.44	21.35 ± 2.31	27.13 ± 1.07

**Fig. 13:**
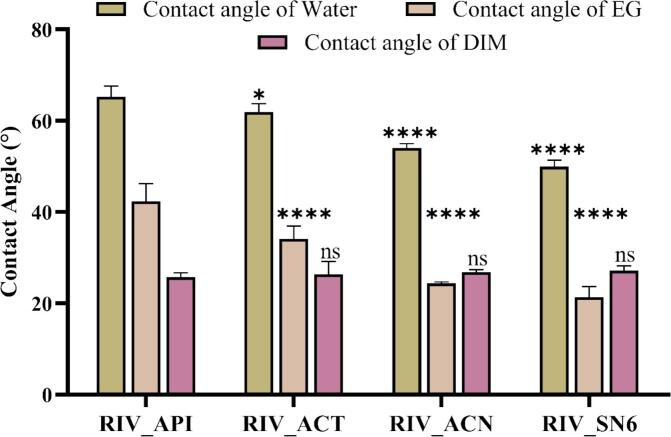
Graphical illustration of the contact angle of RIV_API, and modified crystal samples (RIV_ACT, RIV_ACN, and RIV_SN6). On the basis of two-way ANOVA, and a comparison was done with ‘RIV_API’ which is considered as the control group. The statistical difference here is displayed as ns for non-significance, * if p < 0.05, and **** if p < 0.0001.

The contact angles with the diiodomethane were RIV_API (26.85 ± 0.52), RIV_ACT (25.73 ± 0.94), RIV_ACN (26.32 ± 2.84), and RIV_SN6 (27.13 ± 1.07). The order of their contact angle increment was RIV_ACT < RIV_ACN < RIV_API < RIV_SN6. However, no statistically significant changes in the contact angles of all samples in diiodomethane were observed **(**Fig. 13**)**. The minimum contact angle with water for RIV_SN6 showed more hydrophilicity of the pellet surface, while the minimum contact angle with diiodomethane showed more lipophilicity of the pellet surface. This suggests that the wettability of RIV_SN6 in aqueous solution is greater than that of other samples.

Further, to support the contact angle studies, the surface free energies were calculated **(**Table 6**)** employing the VCG theory (section 2.3.6) from the measured contact angle data.

**Table 6:** Surface free energies (mean value ± SD, n = 3).

	Dispersive surface free energy (mJ/m^2^)	Acidic component (mJ/m^2^)	Polar energy (mJ/m^2^)	Total surface free energy (mJ/m^2^)
RIV_API	45.47 ± 0.18	22.84 ± 0.99	2.88 ± 0.12	48.35 ± 0.14
RIV_ACT	45.88 ± 0.32	16.28 ± 3.43	1.13 ± 0.48	47.01 ± 0.30
RIV_ACN	45.63 ± 0.94	16.80 ± 2.61	1.47 ± 0.65	47.11 ± 0.94
RIV_SN6	45.37 ± 0.38	27.44 ± 1.72	3.08 ± 0.44	48.45 ± 0.38

There was no statistically significant variation was observed in the dispersive surface free energies and total surface free energies among the selecetd samples. However, a significant statistically difference (****p < 0.0001) was seen in the acidic component of RIV_ACN and RIV_SN6 compared to RIV_API. Also, a significant statistically difference of p < 0.05 in the polar energy was observed only in the RIV_SN6 **(**Table 6**)**. The results are in the same line as observed in the contact angle data values. The wettability and discussed surface energies data suggested that the CHM is able to alter the arrangement of polar or non-polar components on the exposed crystal facets. The presence of polar and non-polar components is determined by XPS in the next section 3.11 to support the results observed here.

### 3.11 X-ray photon spectroscopy (XPS) and surface polarity ratio (SPR) analysis

XPS analysis was performed to correlate the contact angle alongwith their respective surface free energies results by determination alongwith quantification of the surface elemental composition. The RIV_API has major elements, including carbon, chlorine, nitrogen, oxygen, and sulfur, of which carbon, chlorine, nitrogen, and sulfur are non-polar components, while oxygen is the only polar component. The atomic concentration (%) of the elements present in the RIV_API, RIV_ACT*,* RIV_ACN*,* and RIV_SN6 are given in Table 7**.** It can be clearly seen that the RIV_SN6 had the highest atomic percentage of oxygen (23.57 %), and the RIV_ACT had the lowest atomic percentage of oxygen (18.65 %). The atomic percentage of oxygen in RIV_API (20.98 %) and RIV_ACN (19.28 %) was lower compared to RIV_SN6. In addition to that, the SPR of the RIV_SN6 was highest (30.22 %) among all other samples. This indicates that the RIV_SN6 surface was having higher polarity than RIV_API, RIV_ACT, and RIV_ACN, and in agreement with the polar energies and acidic component calculated by contact angle (Section 3.10). These results were further supported by the surface chemistry analysis performed through computational simulation.

**Table 7:** Surface elemental constituents (%) of RIV_API, RIV_ACT, RIV_ACN, and RIV_SN6.

Tested Pellet		Atomic concentration (%)				
	Cl 2p	O 1 s	N 1 s	S 2p	C 1 s	(O) / (C + N + S + Cl) (%)
RIV_API	3.25	20.98	7.96	2.50	62.72	27.45
RIV_ACT	2.83	18.65	8.66	2.47	65.30	23.53
RIV_ACN	1.90	19.28	8.93	1.41	65.60	24.77
RIV_SN6	3.50	23.57	8.19	1.84	64.47	30.22

### 3.12 Surface chemistry by computational simulation study

**Fig. 14:**
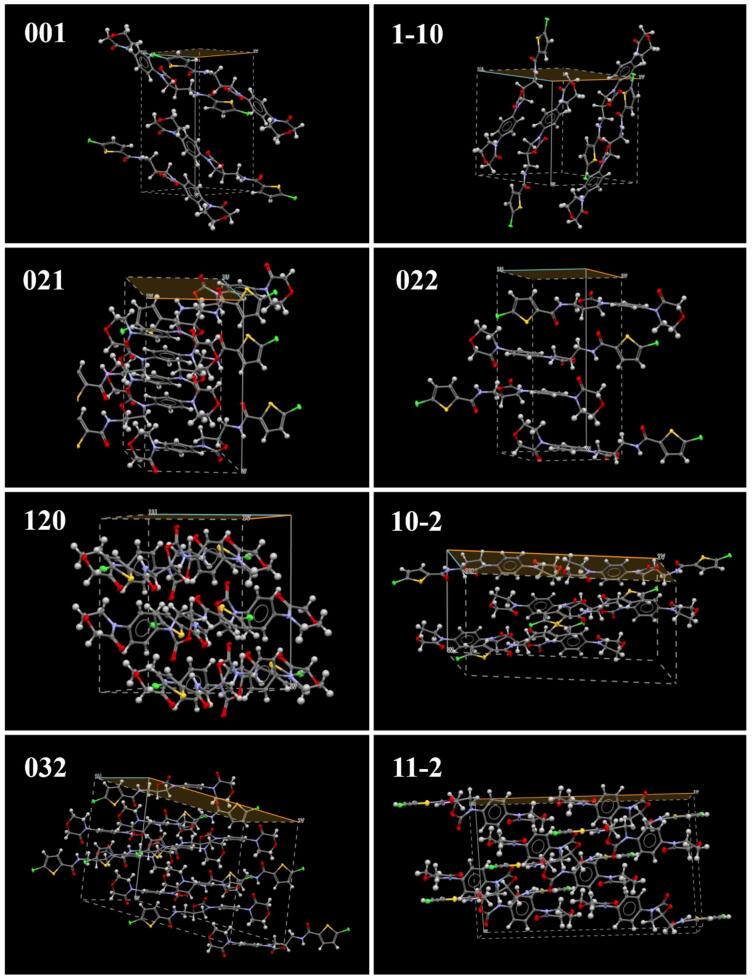
Surface chemistry (anisotropy) of the prominent crystal facets of RIV.

The important facets of RIV were (001), (1–10), (021), (022), (120), (10–2), (032), (11–2) associated with its characteristic peaks of 8.9°, 14.2°, 16.6°, 19.5°, 19.8°, 22.5°, 25.6°, 27.1°, determined through a Mercury software 2022.2.0 (Fig. 14).

The functional groups presented in RIV_API were morpholinone, phenyl, oxazolidinone, amide group, and 5-chloro-thiophene-2-carboxamide. The functional groups that behave as hydrophilic were the amide group, while the lipophilic groups were morpholinone, phenyl, oxazolidinone, and 5-chloro-thiophene-2-carboxamide (Table 8).

**Table 8:** Number of functional groups present in different facets.

Facet	Lipophilic groups				Hydrophilic groups
	Morpholinone	Phenyl	Oxazolidinone	5-chloro-thiophene-2-carboxamide	Amide Group
(001)	**0**	**0**	**0**	**1**	**0**
(1–10)	1	1	0	1	0
(021)	0	0	0	0	2
(022)	0	0	0	0	0
(120)	0	0	0	0	0
(10–2)	**2**	**2**	**2**	**1**	**0**
(032)	**0**	**0**	**1**	**0**	**0**
(11–2)	**1**	**0**	**0**	**0**	**0**

In general, the higher the peak intensity, the higher the facet area exposed in PXRD diffraction, and this results in preferred orientation [77]. Based on higher peak intensity difference and presence of hydrophilic/lipophilic groups, the four facets were selected (001), (10–2), (032), and (11–2) **(**Table S2 in the supplementary information**)**. In the facet (001), the lipophilic group 5-chloro-thiophene-2-carboxamide) was exposed, and the higher intensity was observed in RIV_ACT (24167) compared to other samples, and was considerably lower in RIV_API and RIV_SN6. The presence of the lipophilic group in RIV_ACT seems dominant due to the higher intensity resulting from the larger facet area (001). This suggested that lipophilicity was higher in RIV_ACT and was lower in other samples. In the facet (10–2), the lipophilic group (morpholinone, phenyl, oxazolidinone, and 5-chloro-thiophene-2-carboxamide) was prominently exposed. The higher intensity of facet (10–2) was observed in RIV_ACT (117679) compared to RIV_ACN (59413) > RIV_API (44738) > RIV_SN6 (41009). This suggested that RIV_SN6 was less lipophilic compared to other samples. Similarly, in facet (032), the lipophilic group (oxazolidinone) was majorly exposed, and higher intensity was observed in RIV_ACT (51907) compared to RIV_ACN (45773) > RIV_API (16059) > RIV_SN6 (12231). This also suggested that RIV_SN6 was less lipophilic compared to other samples. In addition to that, in the facet (11–2), the lipophilic group (oxazolidinone) was exposed, and the higher intensity was observed in RIV_ACT (23096) compared to RIV_ACN (22605) > RIV_API (3432) > RIV_SN6 (2133). This also suggested that RIV_SN6 was less lipophilic compared to other samples **(**Table 8**)**. These outcomes support the result from wettability, surface free energies, and XPS analysis. The varied facet areas and the presence of differential hydrophilic/lipophilic groups in a particular facet suggested the possibility of variation in the dissolution rate of the selected samples [9], [49].

### 3.13 Surface area analysis

The surface area of the RIV_API and its modified crystal samples, such as RIV_ACT, RIV_ACN, and RIV_SN6, was determined by the BET using the nitrogen gas adsorption method. Afetr measurements, the surface area of 16.05 m^2^/g of RIV_API was found as the highest among all other samples, while the surface area of RIV_SN6 (12.79 m^2^/g) was the highest when compared to RIV_ACT (1.53 m^2^/g) and RIV_ACN (3.43 m^2^/g). The surface area of the RIV_API was highest due to micrnozed crystals. The differential surface area could cause the difference in dissolution and make it difficult to analyse the impact of the habit modification on their dissolution behaviour. Hence, the impact of surface area due to the variable crystal size on IDR was nullified by passing all the samples through the same sieve (ASTM 100 mesh).

### 3.14 Intrinsic dissolution rate (IDR)

**Fig. 15:**
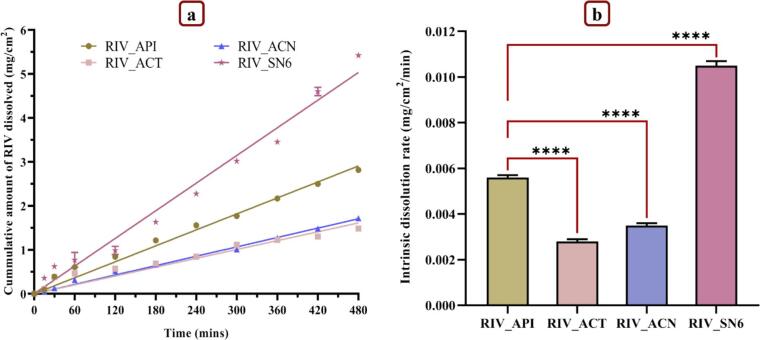
**A)** IDR profiles and **b)** Comparison of IDR of RIV_API, RIV_ACT, RIV_ACN, and RIV_SN6. One-way ANOVA values, and statistical significant differences are indicated by **** if p < 0.0001.

The IDR study was carried out to test the influence of the CHM. The IDR profile of RIV_API and its modified crystal samples is shown in Fig. 15**a.** The RIV_SN6 (0.0105 ± 0.0002 mg/cm^2^/min) showed the highest intrinsic dissolution rate profile compared to RIV_API (0.0056 ± 0.0001 mg/cm^2^/min), RIV_ACT (0.0028 ± 0.0001 mg/cm^2^/min), and RIV_ACN (0.0035 ± 0.0001 mg/cm^2^/min). As seen in 3.10, 3.11, the prominently exposed polar group and hydrophilic component were presented on the particular facet and pellet surface of RIV_SN6, respectively. The statistically significant improvement was only observed in the sonocrystallized RIV_SN6 sample (****p < 0.0001) in comparison to RIV_API. RIV_ACT and RIV_ACN did not exhibit a significant improvement in IDR when compared to RIV_API **(**Fig. 15**b)** due to the fewer polar groups present on the crystal facet. The IDR of all the samples was in agreement as per the results shown in Table 8**.** The improvement in the IDR of the RIV_SN6 can be credited to the prominent exposure of polar functional groups and the increased polar energy, which could lead to better wettability and a higher dissolution rate [28], [49]. This trend observed with IDR was also in line as observed with contact angles and surface free energies **(**Section 3.10**)**. Hence, the improvement in IDR confirmed that sonocrystallization in CHM could be a better alternative to API and CHM without sonocrystallization.

### 3.15 Optimal crystal habit

**Fig. 16:**
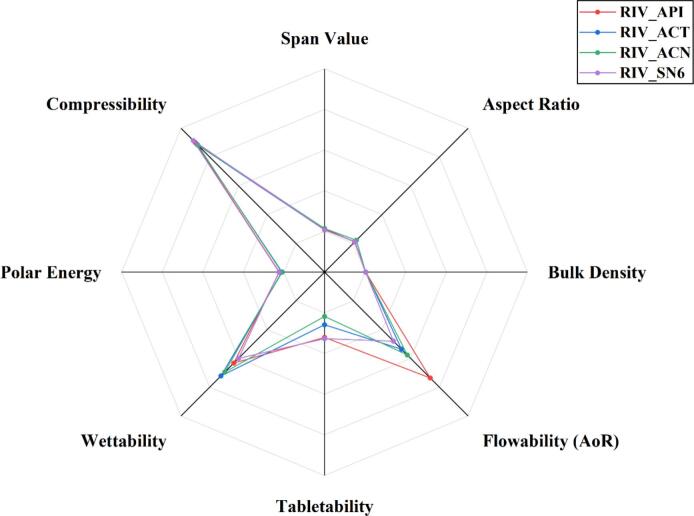
Spider chart illustrating pharmaceutical properties and industrial processability parameters for RIV_API, RIV_ACT, RIV_ACN, and RIV_SN6. Data for this chart are also in Table S**3**.

The spider graph (also known as a radar chart) compares how differently crystal habits affect the various pharmaceutical properties and how easily the material can be processed in the pharmaceutical industry. The spider graph in Fig. 16 shows the impact of modified crystal habit on overall pharmaceutical properties and industrial processability. It is clear that not only does crystal habit improve flowability, but it also significantly improves multiple critical parameters, including the span value (associated with CSD), the aspect ratio (associated with particle shape), the bulk density (associated with crystal packing), the wettability (associated with crystal surface interaction with liquids), polar energy (associated with liquid interaction), compressibility (associated with crystal compressed into tablet), and tabletability (associated with strength and quality of the final tablet). The spider graph was plotted using the data given in Table S**3**. Using a spider graph, the best sample system, RIV_SN6, was selected, which has a tabular-shaped crystal habit.

### 3.16 Stress stability study

The sample of RIV_API and its modified crystal samples subjected to the ICH suggested stress storage conditions are mentioned as RIV_API_S, RIV_ACT_S, RIV_ACN_S, and RIV_SN6_S.

**Solid-state characterization to check polymorphic changes after 1, 2, and 3 months**

**Fig. 17:**
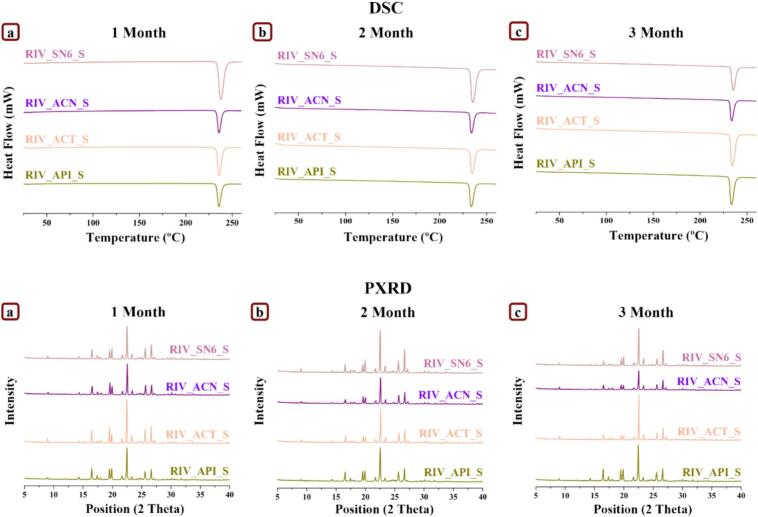
DSC thermograms at **a)** 1**b)** 2, c) 3 months, and PXRD patterns at a) 1, b) 2, c) 3 months of RIV_API and its modified crystals stored at 40 °C and 75 % RH.

The DSC and PXRD were performed for each sample (RIV_API_S, RIV_ACT_S, RIV_ACN_S, and RIV_SN6_S). No polymorphic changes were observed in all the samples kept under stress environment of 40 °C and 75 % RH **(**Fig. 17**)**.

The impact of stress conditions on powder flowability of each sample was examined by Carr’s index and AoR. The flowability of each sample appeared to maintain its original behaviour. In Table 9, no significant difference was observed after completion of 3 months study.

**Table 9:** Micromeritic properties of RIV_API_S and its modified samples stored under stress conditions Mean ± SD (n = 3).

	Bulk Density (g/ml)	Tapped Density (g/ml)	Carr's Index (%)	Angle of Repose (°)
1 Month				
RIV_API_S	0.276 ± 0.002	0.461 ± 0.029	40 ± 3.46	67 ± 1.73
RIV_ACT_S	0.413 ± 0.023	0.535 ± 0.040	23 ± 2.31	42 ± 0.73
RIV_ACN_S	0.381 ± 0.001	0.515 ± 0.015	26 ± 2.00	47 ± 1.32
RIV_SN6_S	0.320 ± 0.003	0.374 ± 0.004	14 ± 0.54	35 ± 0.39
2 Month				
RIV_API_S	0.271 ± 0.001	0.446 ± 0.012	39 ± 1.91	67 ± 0.47
RIV_ACT_S	0.420 ± 0.020	0.544 ± 0.040	23 ± 2.31	41 ± 0.45
RIV_ACN_S	0.383 ± 0.021	0.528 ± 0.037	27 ± 1.15	49 ± 0.86
RIV_SN6_S	0.325 ± 0.004	0.375 ± 0.001	13 ± 1.15	34 ± 0.77
3 Month				
RIV_API_S	0.274 ± 0.002	0.447 ± 0.009	39 ± 1.15	68 ± 0.81
RIV_ACT_S	0.445 ± 0.005	0.571 ± 0.012	22 ± 2.00	42 ± 0.35
RIV_ACN_S	0.379 ± 0.006	0.522 ± 0.016	27 ± 1.15	48 ± 0.63
RIV_SN6_S	0.320 ± 0.005	0.372 ± 0.014	14 ± 2.00	34 ± 0.94

**Fig. 18:**
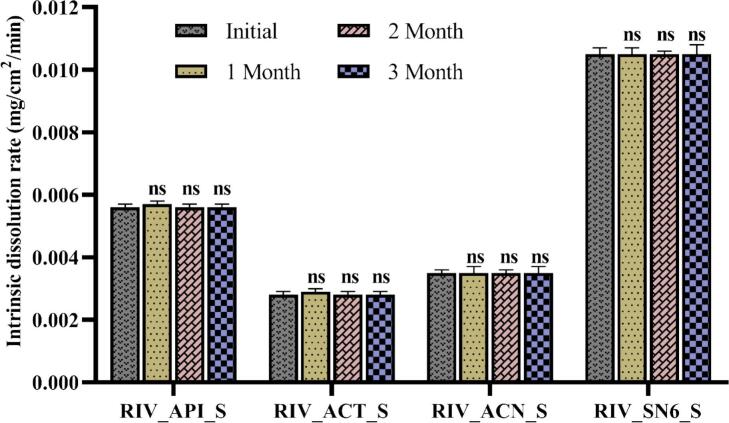
Measurement of intrinsic dissolution rates of RIV_API, RIV_ACT, RIV_ACN, and RIV_SN6 with and without exposure to stress conditions for 1 month, 2 months, and 3 months. On the basis of one-way ANOVA, the statistical differences are reported as non-significant (ns).

Furthermore, an IDR study was also conducted to assess the behaviour of the samples after 3 months of stress stability study. The IDR behavior of the RIV_API_S and its modified crystal samples (RIV_ACT_S, RIV_ACN_S, and RIV_SN6_S) was similar, and no significant changes in the IDR were observed in any of the samples **(**Fig. 18**)** [59]. Hence, based on the stress stability analysis under stress conditions at 40 °C and 75 % RH, the polymorphic state, flowability, and IDR of the original samples were retained.

## 4 Conclusion

In this work, a comprehensive study of the CHM of the RIV was conducted using optimized sonocrystallization. The impact of sonocrystallization on CHM and subsequent pharmaceutical properties was extensively explored. The modified plate-shaped crystal habits (RIV_ACT) and blade-shaped crystal habits (RIV_ACN) were successfully obtained by the cooling evaporation method. The habits produced by the cooling crystallization method still have micromeritic (large size, broad CSD, poor flowability, etc.) and physicochemical (poor compressibility, tabletability, dissolution rate, etc.) properties limitations. With the help of sonocrystallization (especially acaustic cavitation), the crystal habit of the RIV_API was successfully modified from irregular-shaped to tabular-shaped crystals (RIV_SN6). The RIV_SN6 exhibits superior pharmaceutical properties and industrial processability when compared with RIV_ACT and RIV_ACN. The RIV_SN6 exhibits a lower D_90_ with the lower span value. This lower D_90_ value indicates a reduction in crystal size with enhanced specific surface area, and the span value indicates a narrow CSD, thereby improving the packing arrangement of the API. The RIV_SN6 exhibits a tabular-shaped crystal with improved flowability resulting from a modified crystal habit with a lower aspect ratio and a narrow CSD. The sonocrystallized RIV_SN6 showed an improved CTC profile in terms of compressibility and tensile strength, without any tablet deformities up to 500 MPa. The ideal compression at 300 MPa is considered to manufacture the tablet with holding maximum tensile strength. The wettability of the sonocrystallized RIV_SN6 sample was measured using contact angle analysis and found to exhibit higher water wettability as the acidic component and polar energies were higher in RIV_SN6 and were well supported by the surface chemistry study and SPR. The prominence of polar groups and hydrophilic components on the particular facet and pellet surface, respectively, of the sonocrysllized RIV_SN6 sample, resulting in the ∼ 1.88 folds improvement in the IDR. Finally, the sonocrystallized RIV_SN6 samples stored under stress conditions remained stable, with no changes in polymorphic transformation, flowability, or IDR. The current research work highlighted the importance and applicability of sonocrystallization in CHM, thus improving the pharmaceutical properties of the RIV. This work also suggested that sonocrystallization can be used as an alternative to improve the pharmaceutical properties of challenging APIs. This research can be further explored to study the independent contributions of the solvent and ultrasound on CHM. In future work, sonocrystallization can be explored to assess its reliability across other APIs and its scalability for industrial applications.

## CRediT authorship contribution statement

**Maan Singh:** Writing – original draft, Validation, Methodology, Investigation, Formal analysis, Data curation, Conceptualization. **Madhukiran R. Dhondale:** Visualization, Validation, Formal analysis. **Ashish K. Agrawal:** Writing – review & editing. **Dinesh Kumar:** Writing – review & editing, Validation, Supervision, Conceptualization.

## Funding

This research did not receive any specific grant from funding agencies in the public, commercial, or not-for-profit sectors.

## Declaration of competing interest

The authors declare that they have no known competing financial interests or personal relationships that could have appeared to influence the work reported in this paper.
